# Image Processing Techniques for Assessing Contractility in Isolated Adult Cardiac Myocytes

**DOI:** 10.1155/2009/352954

**Published:** 2010-02-24

**Authors:** Carlos Bazan, David Torres Barba, Peter Blomgren, Paul Paolini

**Affiliations:** ^1^Computational Science Research Center, San Diego State University, 5500 Campanile Drive, San Diego, CA 92182-1245, USA; ^2^Department of Mathematics & Statistics, San Diego State University, 5500 Campanile Drive, San Diego, CA 92182-7720, USA; ^3^CardioMyocyte Dynamics Lab, Department of Biology, San Diego State University, 5500 Campanile Drive, San Diego, CA 92182-4614, USA

## Abstract

We describe a computational framework for the comprehensive assessment of contractile responses of enzymatically dissociated adult cardiac myocytes. The proposed methodology comprises the following stages: digital video recording of the contracting cell, edge preserving total variation-based image smoothing, segmentation of the smoothed images, contour extraction from the segmented images, shape representation by Fourier descriptors, and contractility assessment. The different stages are variants of mathematically sound and computationally robust algorithms very well established in the image processing community. The physiologic application of the methodology is evaluated by assessing overall contraction in enzymatically dissociated adult rat cardiocytes. Our results demonstrate the effectiveness of the proposed approach in characterizing the true, two-dimensional, “shortening” in the contraction process of adult cardiocytes. We compare the performance of the proposed method to that of a popular edge detection system in the literature. The proposed method not only provides a more comprehensive assessment of the myocyte contraction process but also can potentially eliminate historical concerns and sources of errors caused by myocyte rotation or translation during contraction. Furthermore, the versatility of the image processing techniques makes the method suitable for determining myocyte shortening in cells that usually bend or move during contraction. The proposed method can be utilized to evaluate changes in contractile behavior resulting from drug intervention, disease modeling, transgeneity, or other common applications to mammalian cardiocytes.

## 1. Introduction

Worldwide, it is estimated that 17.5 million people die of cardiovascular disease (CVD) every year, with an approximate cost of *€*310.23 billion (World Heart Federation. Available at http://www.worldheart.org. Accessed 20090514). Because CVD remains the main cause of death in the world, considerable amounts of resources are devoted to cardiovascular research every year.

The study of cardiocyte contractility has helped unveil the fundamental processes underlying heart function in health and disease [1, 2]. The analysis of cardiocyte mechanics has historically proven an excellent tool in providing relevant information on the excitation-contraction coupling of the heart. Many inotropic factors modulate the contractile behavior of the heart, which can be conveniently studied in enzymatically dissociated (isolated) cardiocytes [2–5]. Researchers commonly measure calcium transient signals, gene and protein expression, and contractility to assess the function and state of these isolated cardiocytes in all their stages [1, 6].

Isolated adult, neonatal, and embryonic cardiocytes from mammalian hearts are widely used in cardiovascular research [2–5]. Adult cardiac ventricular myocytes have been used as analysis tool in cardiovascular research for almost thirty years, and the popularity of this approach is constantly reinforced by the numerous studies published every year [3]. However, during the last decade, the majority of researchers performing long-term (longer than 1 week) studies have favored the use of embryonic and neonatal cardiocytes [5]. Yet, changes in expression of ion channels and contractile protein isoforms during the development of the cardiocytes, pose a problem when making the extrapolation to the fully developed adult cardiocyte. These are strong reasons for researchers to consider using adult cardiocytes when possible [5].

There are several methodologies for assessing the contractility of cardiocytes. The most popular methods include the ones that use laser diffraction techniques [7] and photodiode arrays [8], and those that employ the monitoring of microscopic cell images [9–11]. Among the latter of these is the edge detection method that employs a raster-line to detect changes in myocyte length by sensing left and right cell boundaries using a threshold [10]. This edge detection method is a widespread approach in research involving adult cardiocytes [4, 10, 12, 13]. This method presents some practical difficulties in its implementation. Geometrical and boundary characteristics of adult cardiocytes are the most commonly irregular due to gap junction ends (jagged edge ends), multiple intercalated disks, and variable cell widths, which can potentially complicate the application of the edge detection system on the cardiocyte [4]. Cardiocyte motion can also occur in unexpected directions since cardiocytes will commonly rotate or move vertically—perpendicular to the raster line—depending upon the location or absence of adhesion points [4]. These conditions can lead to complications in the implementation of the edge detection system, and consequently, can result in an inaccurate analysis [4, 12, 13].

## 2. Background and Previous Work

The cardiac myocyte is approximately 25 *μ*m in diameter and about 100 *μ*m in length. It is composed of bundles of myofibrils that contain myofilaments. Myofibrils have distinct, repeating microanatomical units, termed sarcomeres, which are the basic contractile units that make up a myocyte. The region of myofilament structures between two Z-lines is defined as a sarcomere. The Z-line's associated structures are responsible for the lateral alignment of myofibrils and the generation of active contraction in cardiac muscles [14]. The distance between Z-lines—which is equivalent to the sarcomere length—ranges from about 1.6 to 2.2 *μ*m in human hearts. The sarcomere is composed of thick and thin filaments, myosin and actin, respectively. Chemical and physical interactions between the actin and myosin cause the sarcomere length to shorten, allowing the myocyte to contract during the process of excitation-contraction coupling [15].

Contractility can be defined as the intrinsic ability of the heart muscle to generate force and to shorten. At the molecular level, the contractile process originates from the change in concentrations of calcium (Ca^2+^) ions in the myocardial cytosol. Ca^2+^ ions enter through the calcium channel that opens in response to the wave of depolarization that travels along the sarcolemma. These Ca^2+^ ions trigger the release of additional calcium from the sarcoplasmic reticulum, thereby initiating a contraction-relaxation cycle [16].

The need for an accurate method to assess different aspects of a myocyte has led researchers to explore several techniques in order to quantify contractility. Some of these methods are not very popular due to the expensive equipment required, as in the case of the scanning ion conductance microscopy method [6]. This method involves using a distance modulated approach for scanning ion conductance microscopy. It provides a distance control mechanism to image surface sections of contracting myocytes. This technique, combined with laser confocal microscopy, measures myocyte height and local calcium concentration during contractility.

Other methods—such as light diffraction techniques—have been applied to the study of muscle mechanics since the nineteenth century [17]. The reliability of these studies is relatively high, although they are highly dependent upon different factors. These include the temporal resolution of the detection system, sarcomere periodicity values, and other optical artifacts [4]. The sarcomere striation pattern detection method has also been used as a way to quantify contractility. The method can achieve high temporal resolution with the aid of CCD line array detectors and it provides a measure of average sarcomere periodicity from the entire cell or cell regions [18]. One drawback is this method's vulnerability to errors introduced by cell geometry and rotational and translational changes which can occur during contraction [4].

One of the first video-based efforts to measure contraction was performed with the assistance of a device capable of capturing the extent and rate of length shortening of isolated cardiac myocytes [10]. The video-based method uses two tracking points at each end of the myocyte to track edge displacement as the myocyte contracts. The distance between the two edges is measured using edge detection while a record of the data is stored in a separate file. The method generally produces satisfactory results and has been an approved and widely used method for measuring contractile responses of adult myocytes for over twenty years [4, 12].

Several problems have been identified with the application of the video-based edge detection method for measuring adult myocyte contractility [4]. The method can potentially introduce errors to the analysis caused by several factors. The first inconvenience when analyzing myocyte contractility with this method is the need to have the cell positioned parallel to the raster-line. The myocyte should be perfectly positioned in the center of the screen (parallel to the raster-line), and the proper threshold conditions must be set to detect the edges and follow them through a contraction. These threshold conditions are somewhat difficult to set depending upon the characteristics of the cell. The most important source of error that can be potentially introduced during the application of this method is the result of unexpected myocyte movements. Myocytes will often rotate sideways or out of the plane of focus depending upon the presence or absence of adhesion points. The changes in myocyte geometry, dynamic torquing, and rotation can lead to errors in the experiment [2, 4, 12].

We propose a complete computational framework-based on well-established image processing techniques for the assessment of contractility of isolated cardiac myocytes. The proposed methodology is a multi-step process that provides a comprehensive account of the cardiac myocyte contraction process. The proposed method is discussed in the next section.

## 3. Materials and Methods

The proposed computational framework for assessing the contractility in cardiac myocytes comprises the following stages: digital video recording of the contracting cell, edge preserving total variation-based image smoothing, segmentation of the smoothed images, contour extraction from the segmented images, shape representation by Fourier descriptors, and contractility assessment.

Previous to the application of the proposed methodology, the specimen is appropriately prepared as follows. Sprage-Dawley rats purchased from Harlan (CA, USA) were used for this study. The cardiocytes were enzymatically dissociated as described in [19]. After the isolation, the cells remained in the incuvator at least 12 hours in serum-free Medium 199 (GIBCO 12350, Invitrogen Corporation, Carlsbad, CA, USA) before any measurements were performed. Once ready for measurements, the cells were washed twice using serum-free media. Fresh media was then gently added back to the wells. Cardiocytes were platted without Laminin to ensure that the cells were free floating (without adhesion points) in the wells during the digital video recording.

### 3.1. Digital Video Recording

To capture the contraction process of the isolated cardiac myocytes the following procedure was employed. Cells were placed in a chamber mounted on the stage of an inverted microscope (NIKON #ELWD, Nikon Corporation, Tokyo, Japan). Myocytes with obvious sarcolemmal blebs or spontaneous contractions were not used. Only rod-shaped myocytes with clear edges were selected for recording of mechanical properties. The cells were field stimulated with a suprathreshold (50%) voltage at a frequency of 0.33 Hz, for a 3 millisecond duration. The stimulation was performed using a pair of platinum wires placed on opposite sides of the chamber connected to an electrical stimulator (Grass SD9, Grass Technologies, West Warwick, RI, USA). The polarity of the stimulatory electrodes was reversed automatically every 10 stimuli to prevent electrode polarization. Myocyte motion was digitally recorded with a camera (PULNIX TM-1327, JAI PULNiX Inc., San Jose, CA, USA) mounted on the microscope, at a rate of 30 fps. Video files containing the contraction activities were stored for the analysis.

### 3.2. Edge Preserving Total Variation-Based Image Smoothing

We chose a total variation-(TV-)-based method for smoothing isotropic regions while preserving the cell's edges in order to facilitate the segmentation step of the computational framework. Rudin et al. [20] have argued that there are a number of reasons for preferring TV-based image smoothing models over their counterparts. TV-based algorithms are relatively simple to implement and result in minimal ringing (nonoscillatory) while recovering sharp edges (noninvasive). In other words, the TV-norm allows piecewise smooth functions with jumps and is the proper space for the analysis and recovery of discontinuous functions. Also, the TV-based formulations make a priori assumptions about the noise, and therefore they can be tailored to address the specific image restoration problem at hand. Furthermore, empirical evidence suggests that “the human vision favors the *L* ^1^-norm” [21]. In summary, the TV-based formulations seem to be a suitable approach for restoring piecewise continuous functions from noisy and blurry signals. Appendix A provides a more detailed exposition of the original TV-based formulation due to Rudin et al. [22] along with some of the improvements proposed over the years, including the ones by two of the authors of this paper [23–25].

**Figure 1:**
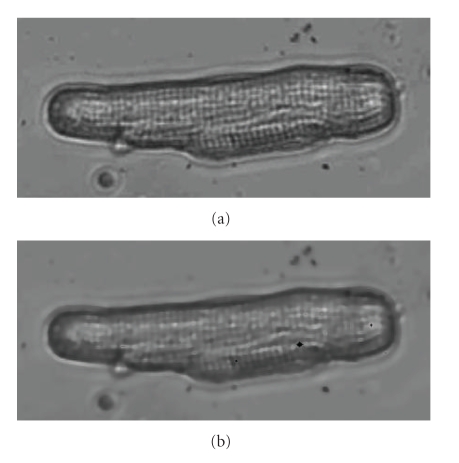
(a) Original image of enzymatically dissociated adult cardiocyte taken from video depicting contractile activity. Contractile activity was recorded using bright light microscopy, while the cell was in a field stimulated chamber. (b) Smoothed image of the same enzymatically dissociated adult cardiocyte during contractile activity, after applying the edge preserving TV-based image smoothing algorithm.

The edge preserving TV-based image smoothing model used in our experiments is given by

(1) $$\begin{array}{c} u_{t}-\left|\nabla u\right|\nabla \cdot \left(\frac{\nabla u}{\left|\nabla u\right|}\right)+\Lambda \left(u-u_{0}\right)=0,\;\text{on}\;\Omega \times \left[\;0,\infty \right),\\ u\left(x,0\right)=u_{0}\left(x\right),\;\text{on}\;\Omega ,\\ \langle g\cdot \nabla u,n\rangle =0,\;\text{on}\;\partial \Omega \times \left(0,\infty \right), \end{array}$$

where *u* and *u*_0_ are the filtered and the observed images, respectively. The dynamic parameter Λ is defined as

(2) $$\Lambda =-\frac{1}{2\sigma^{2}}\nabla u^{\text{T}}\cdot \left(\nabla u-\nabla u_{0}\right),\;\forall t$$

with the approximation to the variance of the noise *σ* ^2^ given by

(3) $$\text{var}\left(\eta^{t+1}\right)=\text{var}\left(u^{t}\right)-\text{var}\left(G_{\sigma }*u^{t}\right),\;\forall t,$$

and the dynamic time step expressed as

(4) $$\delta t=\frac{\varepsilon }{5}+\left(\frac{1}{4}-\frac{\varepsilon }{5}\right)\left(\frac{\max\;\left(\left|\nabla u\right|\right)-\left|\nabla u\right|}{\max\;\left(\left|\nabla u\right|\right)}\right),\;\forall t,$$

with *ε* = 1/255. For more details on this model the reader is referred to [23]. The algorithm to implement the edge preserving total variation-based image smoothing model is given in Appendix B. Figure 1 shows an example of the application of the edge preserving TV-based image smoothing model to a frame depicting an adult myocyte.

### 3.3. Segmentation of the Smoothed Images

**Figure 2:**
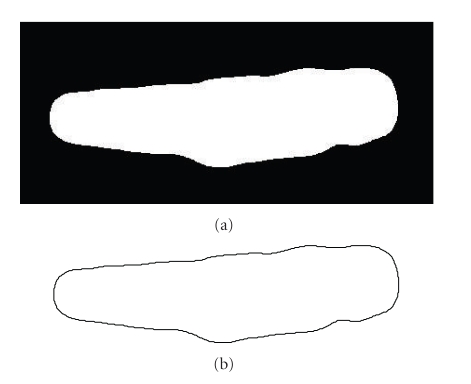
(a) Segmented image of enzymatically dissociated adult myocyte taken from video depicting contractile activity. (b) Final contour extracted from the segmented image of enzymatically dissociated adult myocyte taken from video depicting contractile activity.

Segmentation of an image produces a set of labeled partitions or segments that represent the different components or features. This simplified image allows for an easier extraction of the main contours of the image. In our application this facilitates the identification of the contours of the cell that will permit the assessment of contractility of the cardiac myocyte. Most segmentation algorithms can be used for this purpose. In our application, the speed of execution becomes the principal constraint—because the segmentation of several hundreds of images is required. Thus, we implemented a fast and robust segmentation technique-based on the one presented in [26]. Figure 2-Top shows the segmented image of the cell after applying the segmentation procedure.

### 3.4. Contour Extraction from the Segmented Images

For the contour extraction step of the framework we employ the built-in MATLAB (The MathWorks, Inc., Natick, MA, USA) function that creates a contour plot of image data. We convolve the extracted contour with a Gaussian kernel to eliminate the typical noise produced in the segmentation procedure. We also resample the contour points in all the frames so that they will have the same number of contour points and for this to be equal to 2^*M*^, *M* ∈ *ℕ*. This facilitates the implementation of the discrete Fourier transform algorithm of the next section.

### 3.5. Shape Representation by Fourier Descriptors

Fourier descriptors have been extensively proposed for the purpose of shape recognition, retrieval, classification, and analysis [27–35]. Among the contour-based shape representation methods the ones that have proven more promising for our application are the complex coordinates function and the centroid distance function. In both methods, the Fourier transformed coefficients form the Fourier descriptors of the shape. These Fourier descriptors represent the shape of the object in the frequency domain. The number of Fourier coefficients generated from the Fourier transform is normally large. Nonetheless, the lower frequency Fourier descriptors are the ones that contain the main information about the overall features of the shape. The higher frequency Fourier descriptors, in turn, contain information relative to the finer details of the shape. Therefore, only a relative small number of Fourier descriptors are usually employed to capture the overall features of the shape [36]. For completeness, we present both methods in the following subsections.

### 3.6. Complex Coordinates Function Method

Let *p*(*n*) = (*x*(*n*), *y*(*n*)), for 0 ≤ *n* ≤ *N* − 1, be a discrete function representing the coordinates of a (closed) contour of an image's shape in the Cartesian space, such as the one in Figure 2(b). In the complex plane, we can define this contour as a complex coordinates function *q*, such that

(5) q(n)=x(n)+jy(n).

Then, the Fourier descriptors for the contour of the shape—described by *q*—can be computed using the discrete Fourier transform (DFT) [37]. These Fourier descriptors are the normalized Fourier coefficients

(6) $$Q\left(k\right)=\frac{1}{N}\overset{N-1}{\underset{n=0}{\sum }}q\left(n\right)e^{-j2\pi kn/N},\;k=0,\ldots ,N-1,$$

which represent the discrete contour of the shape in the frequency domain [38, 39].

In order to use the Fourier descriptors as abstract representation of image features in each frame, it is customary to make them invariant to translation, scale, rotation, and their starting point. (In some cases, retaining the step information can be advantageous [38].) For our particular application, we want the Fourier descriptors to change covariantly with the shape of the cell. In other words, we want the Fourier coefficients to capture the contractions of the cell, disregarding only the translation and rotation of the cell. (We also want to make the Fourier descriptors independent of their starting point.)

Translation of the contour function by *τ* ∈ *ℂ* results in a change only to the first Fourier descriptor, *Q*(0). Therefore, by setting *Q*(0) = 0, we move the centroid of the contour onto 0, and make the Fourier descriptors invariant to translations. Invariance with respect to the starting point can be achieved by subtracting the step of the second Fourier descriptor, *φ*_1_ = tan^−1^(*Im* *Q*(1)/*Re* *Q*(1)), weighted by *k*, from the step of all the Fourier descriptors, *Q*(*k*)*e* ^−*jφ*_1_*k*^. Rotation of the contour function by an angle *θ* corresponds to a constant step shift of *θ* in the Fourier descriptors. We can make the Fourier descriptors rotation invariant by computing the magnitude of the Fourier descriptors, |*Q*(*k*)|. The performance of this method is almost identical that of the centroid distance function method explained in the next subsection.

### 3.7. Centroid Distance Function Method

A shape signature—a one-dimensional function derived from the shape boundary coordinates *p*(*n*) = (*x*(*n*), *y*(*n*)), for 0 ≤ *n* ≤ *N* − 1—can be used to generate Fourier descriptors of the shape [39]. Fourier descriptors derived from centroid distance function generally outperform other shape signatures [36, 40]. The centroid distance function of a shape is expressed by the distance of the points on the shape's boundary from the centroid (*x*_*c*_, *y*_*c*_) of the shape

(7) $$\begin{array}{c} r\left(n\right)={\left({\left(x\left(n\right)-x_{c}\right)}^{2}+{\left(y\left(n\right)-y_{c}\right)}^{2}\right)}^{1/2}, \end{array}$$

where

(8) $$\begin{array}{c} x_{c}=\frac{1}{N}\overset{N-1}{\underset{n=0}{\sum }}x\left(n\right),\;\;y_{c}=\frac{1}{N}\overset{N-1}{\underset{n=0}{\sum }}y\left(n\right). \end{array}$$

**Figure 3:**
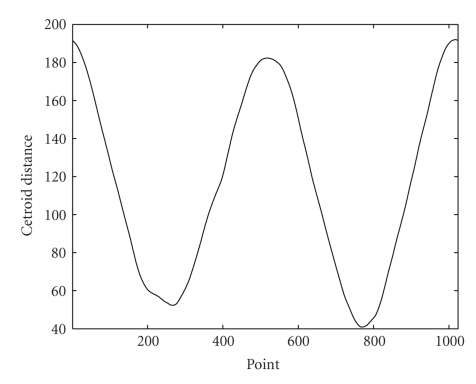
Centroid distance function of the cell shape used in our discussion. The profile of this centroid distance function will be typical in our application.

**Figure 4:**
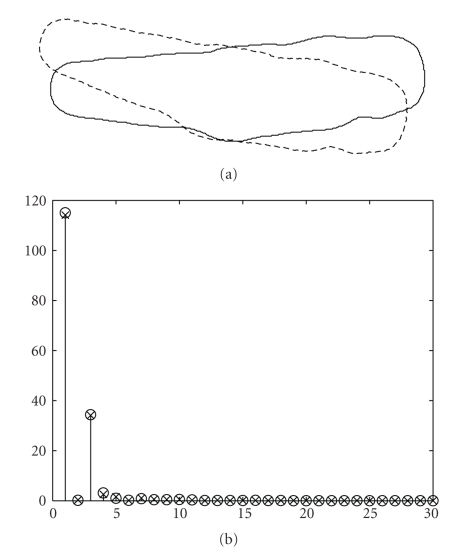
(a) Two identical cell shapes in which one of them has been translated and rotated with respect to the other. (b) first 30 Fourier descriptors for both shapes for the case of translation, rotation, and starting point invariance.

**Figure 5:**
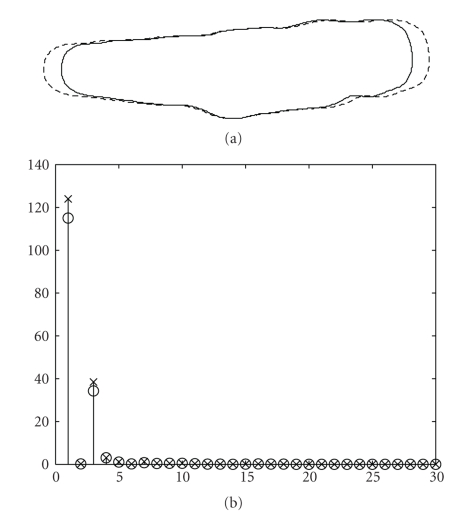
(a) Two cell shapes in which one of them is larger than the other. (b) first 30 Fourier descriptors superimposed. We observe that their Fourier descriptors are able to capture this change in shape size making the Fourier descriptors variant to scale but invariant to translation, rotation, and starting point. The “contraction” of the shape is 8.15% as measured by the Euclidean distance of the Fourier descriptors.

Figure 3 shows the centroid distance function of the cell shape used in the description of these methods. Since function (7) is real-valued, there are only *N*/2 distinct frequencies in the Fourier transform. Thus, only half of the Fourier descriptors will be necessary to describe the shape. Also, by construction, the shape signature *r*(*n*) is invariant to translation. Therefore, we only need to make the Fourier descriptors invariant to rotation and the starting point by identical procedures as in the case of the complex coordinates function method. Figure 4 shows two identical cell shapes, one of which has been translated and rotated with respect to the other. Along with the two shapes, Figure 4 shows both of their first 30 Fourier descriptors superimposed. We observe that both sets of Fourier descriptors match almost perfectly for the case of translation, rotation, and starting point invariance. Figure 5 shows two cell shapes of which one is slightly smaller and has been translated and rotated with respect to the other. Along with the two shapes, Figure 5 shows both of their first 30 Fourier descriptors superimposed. We observe that their Fourier descriptors are able to capture this change in shape size by making the Fourier descriptors variant to scale but invariant to translation, rotation, and starting point.

## 4. Experimental Results

**Figure 6:**
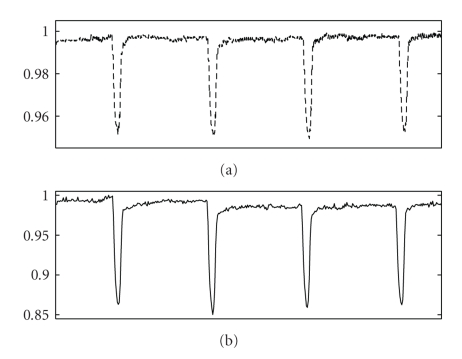
(a) Contraction record of adult enzymatically dissociated rat myocyte under electrical stimulation, analyzed using proposed image analysis-based contractility measuring method. (b) Contraction record of adult enzymatically dissociated rat myocyte under electrical stimulation, analyzed using edge detection system.

**Figure 7:**
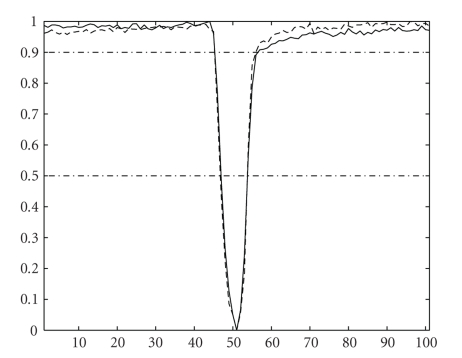
Average of four contractions shown in Figure 6 for both the video-based edge detection system (dashed) and our proposed image analysis-based contractility measuring method (solid). The contractile responses were normalized to fit a desired range. Both records exhibit similar behaviors during the precontraction period, and the contraction to 90% relaxation period, whereas the records show a noticeable difference in the late relaxation period that can be attributed to the two dimensional properties of the proposed image analysis-based contractility measuring method.

We tested the proposed approach by assessing the contractile responses in isolated adult rat cardiocytes, and compared them against the classic raster-line approach [9–11]. We used a sequence of digitized images obtained as previously described for both the proposed method and the raster-line technique. Our results show good qualitative agreement between both methods as far as frequency, pacing, and overall behavior of the contractions are concerned (see Figure 6). Nonetheless, the raster-line method—being a one-dimensional technique—is unable to capture the contraction processes occurring outside its domain of influence. The proposed method, on the other hand, captures the contraction of the cell as a two dimensional event over the entire boundary of the cell. The proposed methodology was also able to capture a slower recovery period than the raster-line method (see Figure 7), which can be attributed to the dimensionality characteristics of both methods. This means that the proposed method is capable of not only assessing the myocyte's length, but also its overall changes in shape and geometry. In other words, it is capable of assessing the myocyte's dimensional changes during contraction while remaining invariant to rotation, translation and starting point.

## 5. Discussion

We presented a complete computational framework for the comprehensive assessment of contractile responses of isolated adult cardiac myocytes. The proposed methodology comprises the following stages: digital video recording of the contracting cell, edge preserving total variation-based image smoothing, segmentation of the smoothed images, contour extraction from the segmented images, shape representation by Fourier descriptors, and contractility measurements. These stages are-based on mathematically sound and computational robust algorithms that are very well established in the image processing community. This makes the methodology easy to understand and implement.

Our results show that this approach—being a two-dimensional technique—is capable of capturing the contractile processes that are otherwise missed by the one-dimensional techniques. This capability makes the method suitable for determining myocyte contraction in cells that usually bend or move during contraction, for example, atrial myocytes and isolated smooth muscle cells, or in cardiac myocytes which develop spatially nonuniform oscillatory contractile activity induced by intracellular calcium fluctuations [10, 41].

Our future work entails the application of the proposed method to analyzing the contractility of myocytes that have been exposed to a drug over a given period of time. We have been investigating one category of the mechanisms that may be responsible for the observed effects on heart cells from a synthetic antidiabetic drug, rosiglitazone (AVANDIA, GlaxoSmithKline, Brentford, UK) of the thiazolidinedione (TZD) family of insulin-sensitizing compounds used in the treatment of type II diabetes. We are anticipating that the proposed method will be an essential tool in that it will complement the analysis of our drug studies, which have been also performed using microarray, Ca^2+^ transient, gene and protein expression measurements. Furthermore, we are in the process of deploying a more sophisticated image acquisition technology that includes a high-speed camera. This will allow for a more in-depth analysis of the contraction process undergone by the cardiac myocyte.

## Figures and Tables

**Table 1:** 

*N* ⇐ 100	number of iterations (%)
*ε* ⇐ 1/255	regularization parameter (%)
*σ* ⇐ 1	Gaussian kernel's width (%)
*d* ⇐ 2	dimensionality of problem (%)
*u*(**x**, 0) ⇐ *u*_0_(**x**), on Ω	set initial condition (%)
∇*u*_0_ ⇐ [(*u*_0_)_*x*_ (*u*_0_)_*y*_]^T^	estimate gradients (%)
*c*_1_ ⇐ corr (*u*, *f*)	performance measure (%)
$\;{\overset{̅}{c}}_{1}⇐\text{corr}\;\;\left(u,u_{0}\right)$	correlation measure (%)
**for** *i* = 1 to *N * **do**	
*u*_*σ*_ = *G*_*σ*_∗*u*	convolve image with Gaussian kernel (%)
*σ* ^2^ = var(*u*) − var(*u*_*σ*_)	estimate variance of the noise (%)
∇*u* ⇐ [*u*_*x*_ *u*_*y*_]^T^	estimate gradients (%)
$\left|\nabla u\right|⇐\sqrt{u_{x}^{2}+u_{y}^{2}}$	magnitude of the gradients (%)
$g⇐\frac{1}{\sqrt{u_{x}^{2}+u_{y}^{2}+ɛ}}$	diffusivity function (%)
〈*g* · ∇*u*, **n**〉 ⇐ 0, on ∂Ω	set boundary conditions (%)
$\Lambda =-\frac{1}{2\sigma^{2}}\left[u_{x}\left(u_{x}-{\left(u_{0}\right)}_{x}\right)+u_{y}\left(u_{y}-{\left(u_{0}\right)}_{y}\right)\right]$	forcing term parameter (%)
*ϕ* = |∇*u*|∇ · (*g* · ∇*u*) − Λ(*u* − *u*_0_)	diffusion term (%)
$\delta t\left(x\right)=\frac{\varepsilon }{5}+\left(\frac{1}{2d}-\frac{\varepsilon }{5}\right)\left(\frac{\max\;\left(\left|\nabla u\right|\right)-\left|\nabla u\right|}{\max\;\left(\left|\nabla u\right|\right)}\right)$	time-step (%)
*u* ⇐ *u* + *δ* *t* *ϕ*	evolve the image (%)
*c*_*i*+1_ ⇐ corr (*u*, *f*)	update performance measure (%)
${\overset{̅}{c}}_{i+1}⇐\text{corr}\;\left(u,u_{0}\right)$	update correlation measure (%)
${\hat{c}}_{i+1}⇐\partial^{2}{\overset{̅}{c}}_{i+1}$	stopping criterion (%)
**if** $\partial {\hat{c}}_{i+1}\leq 0\;$ **then**	
*v* = *u*	save best image if condition is met (%)
** end if**	
**end for**	

## Appendices

### A. Total Variation-Based Models in Image Processing

Rudin et al. [22] proposed removing noise from images by minimizing the TV norm of the estimated solution. They derived a constrained minimization algorithm as a time-dependent nonlinear PDE, where the constraints are determined by the noise statistics. They stated that the space of bounded (total) variation (BV) is the proper class for many basic image processing tasks. Thus, given a noisy image *u*_0_ = *f* + *η*, where the true image *f* has been perturbed by additive white noise *η*, the restored image *u* ≈ *f* is the solution of

(A.1) $$\underset{u\in \text{BV}\left(\Omega \right)}{\min\;}\text{TV}\left(u\right)=\underset{u\in \text{BV}\left(\Omega \right)}{\min\;}\int_{\Omega }\left|\nabla u\right|dx,$$

subject to the following constraints involving the noise:

(A.2) $$\begin{array}{c} \frac{1}{2}\int_{\;\Omega }{\left(u-u_{0}\right)}^{2}dx=\frac{1}{2}\left|\Omega \right|\sigma^{2},\\ \frac{1}{\left|\Omega \right|}\int_{\Omega }u_{0}\;dx\frac{1}{\left|\Omega \right|}\int_{\Omega }u\;dx, \end{array}$$

where |Ω| represents the area of the image. The first constraint uses a priori information that the standard deviation of the noise is *σ*, while the second constraint assumes that the noise has zero mean. (It can also be shown that (A.1) and (A.2) imply that the noise is normally distributed [42].) The TV-norm does not penalize discontinuities in *u*, and; therefore, it allows the recovery of the edges in the observed image *u*_0_.

To solve this minimization problem we would usually solve its associated Euler-Lagrange equation, namely,

(A.3) $$\begin{array}{c} -\nabla \cdot \left(\frac{\nabla u}{\left|\nabla u\right|}\right)+\lambda \left(u-u_{0}\right)=0, \end{array}$$

on a closed domain Ω, and subject to homogeneous Neumann boundary conditions on the boundary ∂Ω. The solution procedure proposed in [22] employs a parabolic equation with time as an evolution (scale) parameter, or equivalently, the gradient descent method, that is,

(A.4) $$u_{t}-\nabla \cdot \left(\frac{\nabla u}{\left|\nabla u\right|}\right)+\lambda \left(u-u_{0}\right)=0,$$

for *t* > 0, on a closed domain Ω, with the observed image as initial condition, *u*(**x**, 0) = *u*_0_(**x**), and homogeneous Neumann boundary conditions, 〈*g* · ∇*u*, **n**〉 = 0, on the boundary ∂Ω. For the parameter *λ*, they suggested a dynamic value *λ*(*t*) estimated by Rosen's gradient-projection method, which as *t*→*∞* converges to

(A.5) $$\lambda =-\frac{1}{2\left|\Omega \right|\sigma^{2}}\int_{\;\Omega }\;\left[\left|\nabla u\right|-\frac{\nabla u_{0}^{T}\nabla u}{\left|\nabla u\right|}\right]dx.$$

Existence and uniqueness results for this nonlinear PDE have been obtained by Lions et al. [43]. Other successful implementations of this minimization problem include the second order cone programming [44], convex programming [45], duality [46], and a fast and exact minimization method-based on graph cuts [47, 48].

Nonetheless, the Rudin-Osher-Fatemi model, in its original form, presents several practical challenges [49]. This evolution scheme is not trivial to implement since it is highly nonlinear and not well-posed [50]. When the scheme converges it does so at a linear rate. It can also run into trouble when |∇*u*| → 0 beyond machine accuracy. In practice, it is very common to use a slightly modified version of the TV-norm [49]:

(A.6) $$\int_{\;\Omega }{\left({\left|\nabla u\right|}^{2}+ɛ\right)}^{1/2}\;dx,$$

where *ε* is a small positive number which “smoothes out the corner” at |∇*u*| = 0. The two other practical (observable) limitations presented by the Rudin-Osher-Fatemi original model are the loss of contrast [51, 52] and the “staircase” effect, that is, a strong preference for piecewise constant patches [53, 54]. The Rudin-Osher-Fatemi model has been extensively studied and improved upon by many scientists [20, 49–52, 54–65].

Marquina and Osher [54] proposed a different version of the transient parabolic equation that helps speed up the convergence of the time-marching scheme. The new evolution equation is

(A.7) $$u_{t}-\left|\nabla u\right|\nabla \cdot \left(\frac{\nabla u}{\left|\nabla u\right|}\right)+\left|\nabla u\right|\lambda G_{\sigma }*\left(G_{\sigma }*u-u_{0}\right)=0,$$

for *t* > 0, on a closed domain Ω, with the observed image as initial condition, *u*(**x**, 0) = *u*_0_(**x**), and homogeneous Neumann boundary conditions, 〈*g* · ∇*u*, **n**〉 = 0, on the boundary ∂Ω, and where *G*_*σ*_ is a blurring operator (Gaussian kernel). This approach fixes the staircase problem of the original scheme and is used for the removal of both blur and noise.

Bazán and Blomgren [24] implemented a variation of the Blomgren et al. [55] version of the Rudin [22] Euler-Lagrange equation as modified by Marquina and Osher [54]. They referred to this approach as Parameter-Free Adaptive Total Variation-Based Noise Removal and Edge Strengthening Model. For our current application, we will use this method for estimating the unknown noise level and their pixel-wise definition for the parameter *λ*. Given the assumption that the image has been perturbed by additive white noise, *u*_0_ = *f* + *η*, and that this noise is independent from the signal, the variance of the noisy image must be equal to the sum of the variance of the true image and the variance of the noise, that is, var(*u*_0_) = var(*G*_*σ*_∗*u*_0_) + var(*η*). Here, the variance of the (unknown) true image is approximated by the variance of the convolved noisy image with a Gaussian kernel of width *σ* = 1. This parameter will be updated at every iteration which provides a positive effect. For the parameter *λ* they proposed a variation of the method suggested in [22]. Instead of integrating (or summing) over the domain Ω, they assumed a pixel-wise parameter as

(A.8) $$\Lambda \equiv \left|\nabla u\right|\lambda =-\frac{1}{2\sigma^{2}}\left[u_{x}\left(u_{x}-{\left(u_{0}\right)}_{x}\right)+u_{y}\left(u_{y}-{\left(u_{0}\right)}_{y}\right)\right].$$

For a detailed explanation of the attributes of the dynamic parameter Λ, the reader can consult [23].

Due to the high nonlinearity of the TV-based models, to ensure stability, the required time step is very small. Song [50] has shown that the CFL condition for the Rudin-Osher-Fatemi model is *δ* *t*/*δ* *x* ^2^ ⩽ *c*|∇*u*|, with *c* > 0. He has also shown that the CFL condition for the Marquina-Osher model is *δ* *t*/*δ* *x* ^2^ ⩽ *c*, with *c* > 0. As a rule of thumb, Gilboa [66] has suggested (assuming *δ* **x** = 1) setting the value of *δ* *t* = *ε*/5, where *ɛ* is the regularization constant used in (A.6). Weickert et al. [67] have shown that for explicit discretization schemes, the stability condition for the Perona-Malik-type models (assuming *δ* **x** = 1 and for  all  *s* : *g*(*s*) ⩽ 1) is *δ* *t* < 1/2*d*, with *d* being the number of dimensions of the data. In his dissertation, Bazán [23] argued that since the explicit discretization schemes used in the TV-based models produce updates of the following form:

(A.9) $$u^{t+1}=u^{t}+\delta tF\left(u^{t},\nabla u^{t},u_{0},\nabla u_{0},\lambda \right),$$

then, in practice, the smaller the time-step, the slower the restoration process. He used the aforementioned findings to devise an “adaptive time-step” *δ* *t*(**x**, *t*), which does not only make the TV-based schemes more stable (smooth), but also speeds up the restoration process. The proposed adaptive time-step is

(A.10) $$\delta t\left(x\right)=\frac{\varepsilon }{5}+\left(\frac{1}{2d}-\frac{\varepsilon }{5}\right)\left(\frac{\max\;\left(\left|\nabla u\right|\right)-\left|\nabla u\right|}{\max\;\left(\left|\nabla u\right|\right)}\right),\;\forall t,$$

where, as before, *ɛ* is the regularization constant used in (A.6), and *d* is the number of dimensions of the data. For a detailed description of the characteristics of the adaptive time-step the reader is referred to [23].

### B. Numerical Implementation of the Edge Preserving Total Variation-Based Image Smoothing

The algorithm to implement the parameter-free adaptive TV-based noise removal and edge strengthening model is in Table 1.
